# Whole‐Exome Sequencing Identifies an Intronic Cryptic Splice Site in *SERPINF1* Causing Osteogenesis Imperfecta Type VI

**DOI:** 10.1002/jbm4.10044

**Published:** 2018-04-16

**Authors:** Zixue Jin, Lindsay C Burrage, Ming‐Ming Jiang, Yi‐Chien Lee, Terry Bertin, Yuqing Chen, Alyssa Tran, Richard A Gibbs, Shalini Jhangiani, V Reid Sutton, Frank Rauch, Brendan Lee, Mahim Jain

**Affiliations:** ^1^ Department of Molecular and Human Genetics Baylor College of Medicine Houston TX USA; ^2^ Texas Children's Hospital Houston TX USA; ^3^ Human Genome Sequencing Center, Baylor College of Medicine Houston TX USA; ^4^ Shriners Hospital for Children and McGill University Montreal Canada; ^5^ Kennedy Krieger Institute Baltimore MD USA

**Keywords:** OSTEOGENESIS IMPERFECTA TYPE VI, SPLICE VARIANT, INTRONIC VARIANT, GENETIC RESEARCH, MATRIX MINERALIZATION

## Abstract

The heritable disorder osteogenesis imperfecta (OI) is characterized by bone fragility and low bone mass. OI type VI is an autosomal recessive form of the disorder with moderate to severe bone fragility. OI type VI is caused by mutations in the serpin peptidase inhibitor, clade F, member 1 (*SERPINF1*), the gene coding for pigment epithelium‐derived factor (PEDF). Here, we report a patient with OI type VI caused by a novel homozygous intronic variant in *SERPINF1* identified by whole‐exome sequencing (WES). The mutation was not identified using a low bone mass gene panel based on next‐generation sequencing. This variant creates a novel consensus splice donor site (AGGC to AGGT) in intron 4. Analysis of cDNA generated from fibroblasts revealed retention of a 32‐bp intronic fragment between exons 4 and 5 in the cDNA, a result of alternative splicing from the novel splice‐donor site. As a result, the aberrant insertion of this intronic fragment generated a frameshift pathogenic variant and induced nonsense‐mediated decay. Furthermore, gene expression by quantitative PCR showed *SERPINF1* expression was dramatically reduced in patient fibroblasts, and PEDF level was also significantly reduced in the patient's plasma. In conclusion, we report a novel homozygous variant that generates an alternative splice‐donor in intron 4 of *SERPINF1* which gives rise to severe bone fragility. The work also demonstrates clinical utility of WES analysis, and consideration of noncoding variants, in the diagnostic setting of rare bone diseases. © 2018 The Authors. *JBMR Plus* is published by Wiley Periodicals, Inc. on behalf of American Society for Bone and Mineral Research.

## Introduction

Osteogenesis imperfecta (OI) is a genetic disorder characterized by low bone density and bone fragility. Approximately 90% of individuals with OI have heterozygous pathogenic variants in the bone matrix protein collagen type I, *COL1A1* and *COL1A2*,^1^, ^2^, ^3^, ^4^ that are de novo or inherited in an autosomal dominant pattern from an affected parent. OI type VI is a recessive form with moderate to severe bone fragility. Bone histology in OI type VI shows a mineralization defect and a large amount of unmineralized osteoid.^5^ Bone biopsies from individuals with OI type VI have a “fish‐scale” appearance, though this finding can be seen in other pathogenic states.^5^, ^6^ OI type VI is caused by pathogenic variants in the serpin peptidase inhibitor, clade F, member 1 (*SERPINF1*), the gene coding for pigment epithelium‐derived factor (PEDF).^6^, ^7^, ^8^, ^9^ A missense variant in *IFITM5* has also been reported in one case.^10^ More than 25 disease‐causing *SERPINF1* variants have been reported including frameshift, nonsense variants, and in‐frame insertion or deletion variants, which caused reduced expression or altered intracellular localization of PEDF (Osteogenesis Imperfecta Variant Database, Leiden University Medical Center, Leiden, The Netherlands; https://oi.gene.le.ac.uk/home.php).^11^, ^12^, ^13^, ^14^, ^15^ Most of the reported variants are exonic and located in coding regions of *SERPINF1*.^16^ Recently, a novel splice site in intron 6 of *SERPINF1* (c.787–10C>G) has been identified in two individuals with OI type VI.^17^ The variant led to an in‐frame addition of three amino acids to PEDF and a decreased secretion of PEDF.^17^ In the present study, we report a novel and deeper pathogenic intronic variant of *SERPINF1* discovered by whole‐exome sequencing (WES) in a young man with OI type VI. This intronic variant introduces a novel splice‐donor site and results in the aberrant insertion of a 32‐bp intronic fragment that produces a frameshift variant and induces nonsense‐mediated decay.

## Materials and Methods

### Human subjects

This study was approved by the Institutional Review Board at Baylor College of Medicine. The family provided informed consent for study procedures and for publication of medical information.

### WES and filtering

Exome sequencing was performed as described.^18^, ^19^ Briefly, genomic DNA was isolated from peripheral blood monocytes. Exomes were captured on Nimblegen's Baylor VCRome library (Roche NimbleGen, Madison, WI, USA), and sequencing was performed on the Illumina HiSeq 2000 platform (Illumina). Trio exome sequencing was completed, and resulting data were aligned and processed per the MERCURY pipeline.^20^ We performed joint calling using PLATYPUS v 0.7.9.1^21^ and filtered resulting variants against public databases, including the 1000 Genomes phase 3^22^ and the ExAC database version 0.3,^23^ to retain variants that were found at an allele frequency of less than 1%. We performed annotation using Annovar^24^ and retained all rare annotated variants, including intronic variants. We reviewed all variants within *SERPINF1* and *IFITM5* loci.

### RNA extraction and real‐time qPCR

Total RNA was extracted from fibroblasts derived from the patient and a control sample using Trizol reagent. The Superscript III First Strand RT‐PCR kit was used to synthesize cDNA according to the manufacturer's protocol (Invitrogen). Real‐time qPCR was performed on LightCycler instrument (Roche) with β‐actin as internal control.

### PEDF level in plasma

We measured plasma PEDF level by ELISA, using a kit from BioProductsMD (Middletown, MD, USA). Briefly, plasma was stored at −80°C before use. The plasma was thawed on ice and treated with 8M urea. Then, the ELISA was performed according to the manufacturer's protocol.

## Results

### Clinical findings

**Figure 1:**
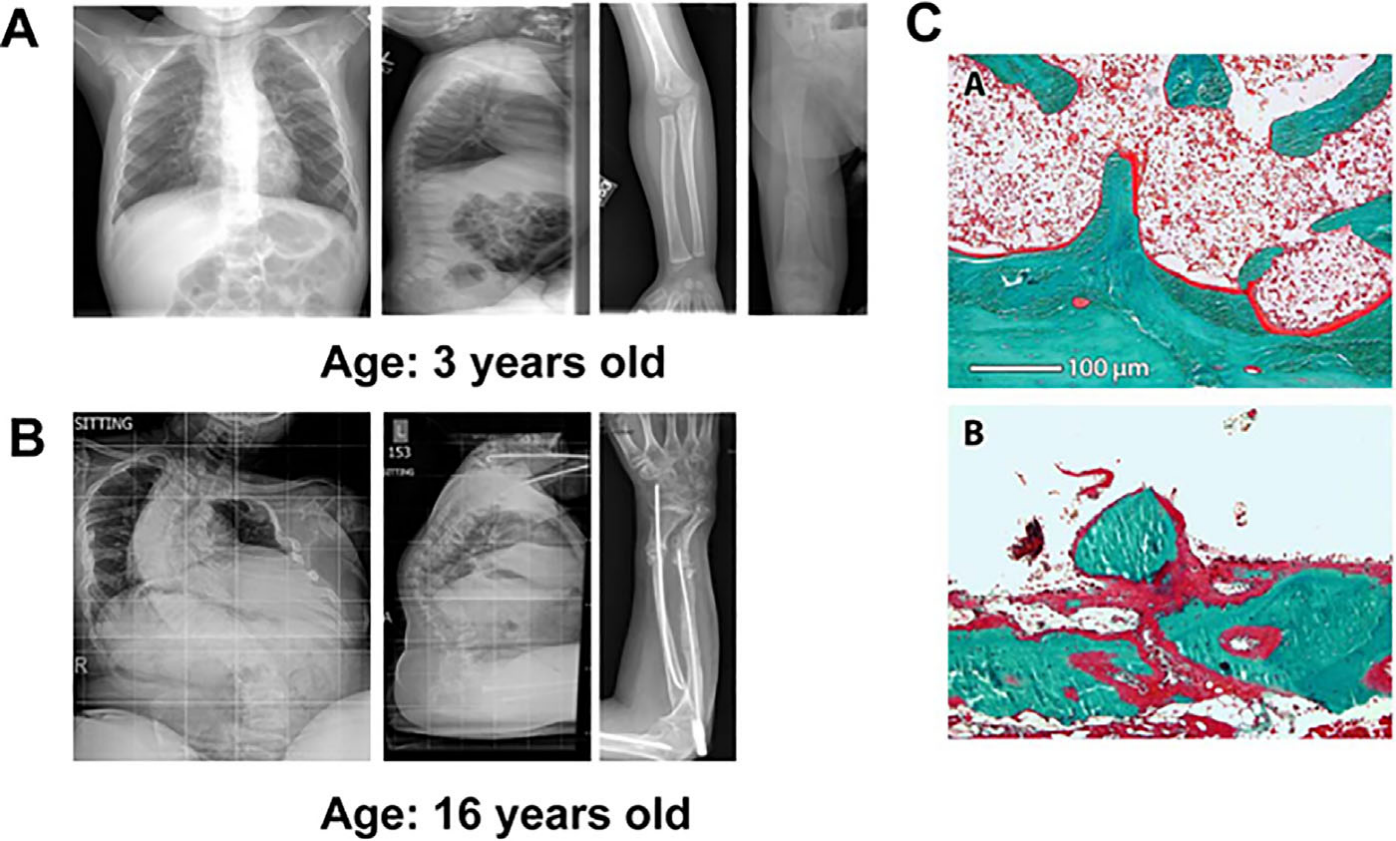
Radiographs and histomorphometry. (*A*) Radiograph showing femoral fracture and scoliosis (3 years old). (*B*) Radiograph at 16 years of age demonstrating progression to severe scoliosis. (*C*) Iliac bone biopsy specimen (Goldner trichrome staining; osteoid shown in red, mineralized bone in green) of (a) a control (female, 2.1 years) and (b) the proband with OI type VI (2.6 years).

The proband was born full term after an uncomplicated pregnancy to unaffected parents. His early development was normal, and he had his first fracture (left femur) when he started walking at 10 months of age. His next fracture was a right femoral fracture at the age of 18 months that occurred while sitting. He subsequently had two additional femoral fractures prior to age of 21 months. At the age of 3 years, he had a bone density scan (dual‐energy X‐ray absorptiometry [DXA]) that demonstrated a *Z* score of −5.4 (lumbar spine). Collagen studies were normal, and he has been treated with intravenous bisphosphonate therapy since 2 to 3 years of age. Currently, he is 18 years of age, and he has short stature, bowed extremities, severe scoliosis, and mild joint laxity. He does not have dentinogenesis imperfecta nor does he have a blue‐gray color of his sclerae. He is otherwise healthy and cognitively normal. He has had numerous fractures, and his most recent DXA scan revealed a *Z* score of −2.3 at the lumbar spine at age 13 years of age. He has had placement of Fassier‐Duval rods in both femurs. He has also had rod placement in both humeri and in both forearms. He uses a wheelchair for mobility. Representative X‐rays at 3 years of age and at 16 years of age are provided in Fig. 1 *A* and *B*, respectively. Analysis of a transiliac bone biopsy at 2.6 years of age, made a clinical diagnosis of OI type VI, as it showed characteristic features of the condition. It showed hyperosteocytosis, large osteocytic lacunae, a “fish‐scale appearance,” and an increased amount of unmineralized osteoid (Fig. 1 *C*, Table 1), as well as a low amount of trabecular bone (Table 1).^25^

**Table 1:** Results of Iliac Bone Histomorphometry in the Proband

Iliac biopsy	OI individual	Controls^25^, ^a^
Bone volume/tissue volume (%)	9.8	17.7 ± 2.6
Trabecular thickness (µm)	91	101 ± 11
Trabecular number(/mm)	1.1	1.8 ± 0.3
Osteoid thickness (µm)	10.5	5.8 ± 1.4
Osteoid surface/bone surface (%)	87	34 ± 7
Osteoid volume/bone volume (%)	19.5	4.0 ± 1.2

^a^ Control histomorphometric parameters are from 1.5 to 6.9 years of age (6 male, 4 female).

### *SERPINF 1* gene analysis

**Figure 2:**
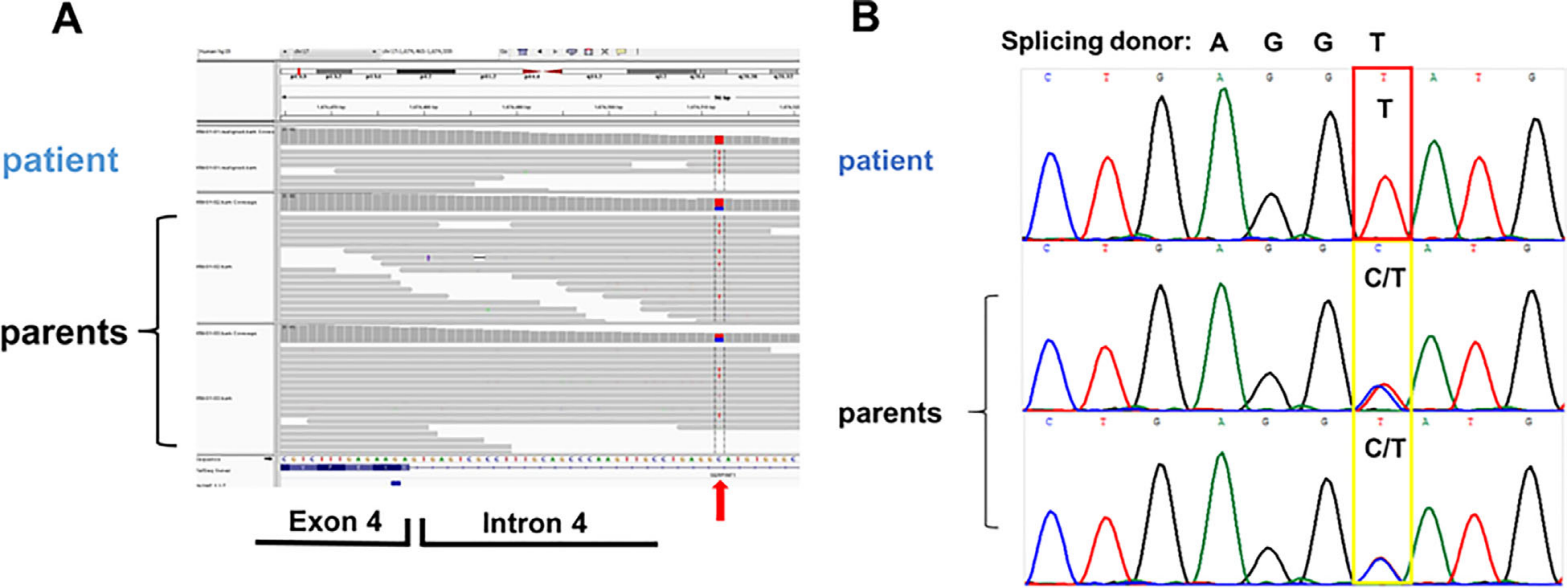
DNA sequence results of *SERPINF1*. (*A*) Analysis of whole exome sequencing from the parent‐child trio identified a novel intronic variant. (*B*) Sanger sequencing confirmed the homozygosity of the patient and heterozygosity of the parents.

A low bone mass gene panel (next‐generation sequencing) that included *SERPINF1*, *IFITM5*, and 21 other genes implicated in low bone density did not reveal causative pathogenic variants.^26^ Thus, we performed WES for the parent‐child trio. We did not observe protein coding variants in *SERPINF1* or variants in the 5′ untranslated region (UTR) or coding regions of *IFITM5*. However, he was found to have a novel homozygous variant (ENST00000254722.4:c.439+34C>T) in *SERPINF1*, with both parents being heterozygous (Fig. 2 *A*). This variant creates a novel consensus splice donor site (AGGC to AGGT) in intron 4 of *SERPINF1* (Fig. 2 *A*), which is after a nonconsensus splice donor at the exon 4/intron 4 boundary (GAGT). The variant is not observed in public databases.^22^, ^23^ We further confirmed the homozygosity of the patient and heterozygosity of both parents by Sanger sequencing (Fig. 2 *B*).

### *SERPINF1* cDNA analysis

**Figure 3:**
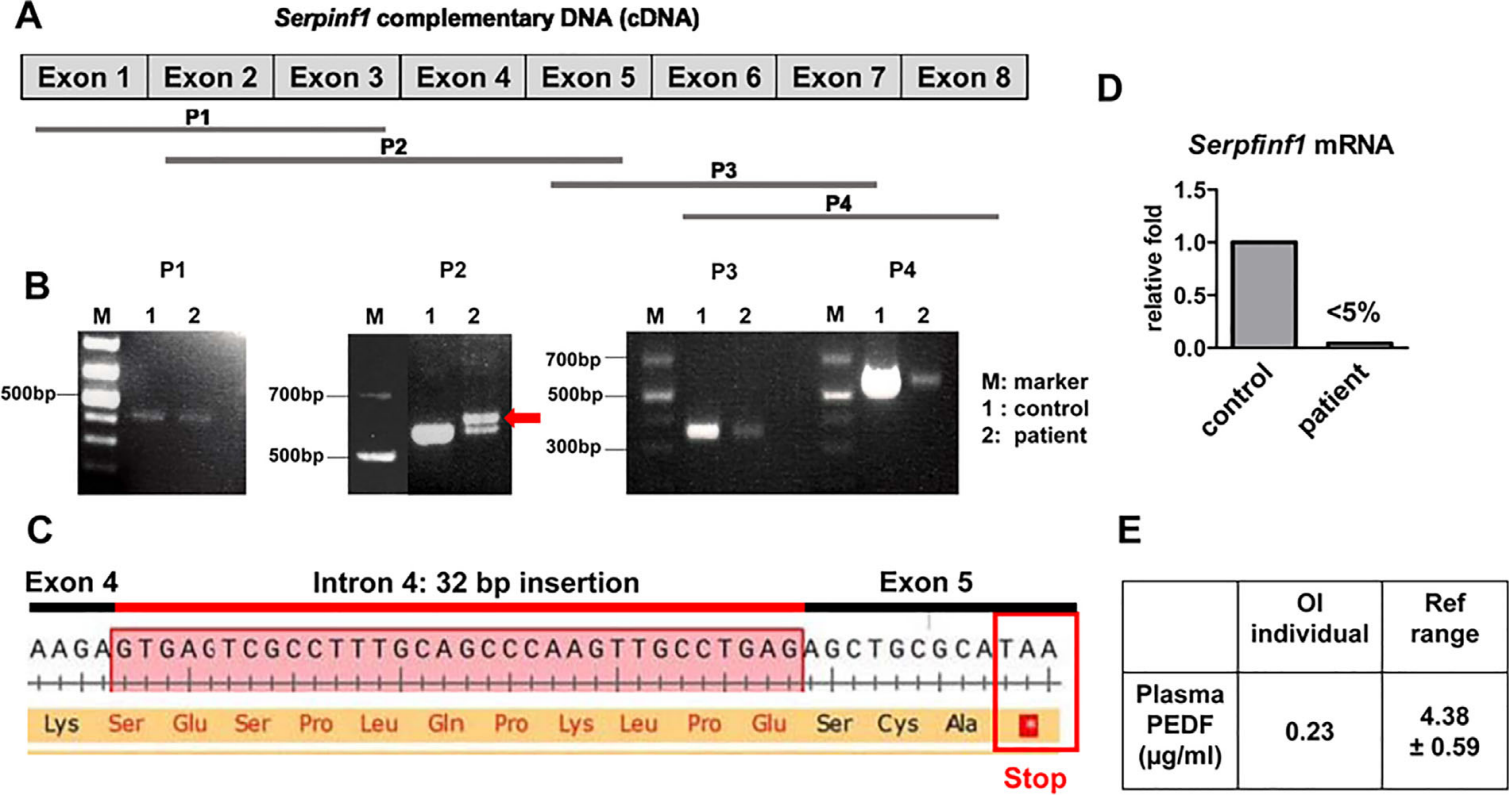
cDNA analysis of *SERPINF1*. (*A*) Total RNAs were isolated from control and subject's fibroblasts, reverse‐transcribed, and PCR‐amplified to generate the indicated fragment. (*B*) Aberrant cDNA sequence with an insertion of 32‐bp intron fragment. (*C*) Retention of 32‐bp intron creates a premature stop codon. (*D*) Expression of *SERPINF1* was dramatically decreased as shown by real‐time PCR. (*E*) Systemic PEDF level was significantly decreased as shown by ELISA.

**Figure 4:**
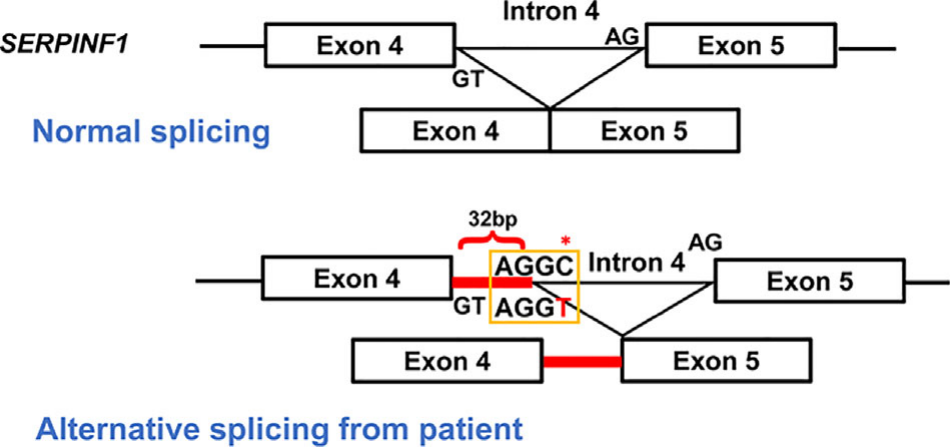
Processing of *SERPINF1* pre‐mRNA transcript during normal splicing and alternative splicing from the proband with introduction of a splice‐donor site in intron 4.

The detection of an intronic splice donor variant prompted us to analyze the cDNA as well as the expression level of *SERPINF1* in the patient. We synthesized cDNA from fibroblasts of the patient and control sample, and we designed a series of primers to amplify cDNA fragments (Fig. 3 *A*). The primer sequences are shown in Supporting Table  1. We detected an extra band of PCR product (indicated in red arrow) using primers amplifying cDNA between exon 2 and exon 5 in patient cDNA compared with normal control (Fig. 3 *A*). To determine the sequence of the two bands from patient cDNA, we cloned and sequenced the PCR amplicons. Besides the normal sequence (the band of the same size of control), the larger amplicon had a 32‐bp intronic sequence retention between exon 4 and exon 5 (Fig. 3 *B*). The 32‐bp fragment is proximal to the 5′ splicing donor site in intron 4. Here we confirmed our hypothesis that the intronic variant near exon 4 introduced a new splice donor site in the patient. As a result, the retention of the 32‐bp fragment created a premature stop codon (Fig. 3 *C*). To further confirm that the new splice donor variant is the disease‐causing variant, we tested *SERPINF1* expression and showed that the mRNA expression of *SERPINF1* was dramatically decreased to about 5% in patient fibroblasts (Fig. 3 *D*). Plasma level of PEDF was also dramatically reduced to 0.23 µg/mL in the patient as compared to the range of 4.38 ± 0.59 µg/mL in unaffected populations^27^ (Fig. 3 *E*). These data suggest that the identified intronic variant is a hypomorphic mutation, given that there is a small amount of PEDF (about 5%) in the plasma. Because of the alternative splicing, 32 nucleotides from intron 4 were retained between exon 4 and 5 producing a frameshift variant and nonsense‐mediated decay (Fig. 4). The loss of mRNA, results in systemic loss of PEDF leading to the phenotype.

## Discussion

The majority of previously reported *SERPINF1* pathogenic variants have been described in coding regions. Recently, Ward and colleagues^17^ reported a homozygous variant in intron 6 of SERPINF1 (c.787–10C>G). Here, we report a novel and deeper intronic single‐nucleotide variant in *SERPINF1* generating a splice‐donor site that gives rise to severe bone fragility. Exonic or intronic variants affecting mRNA splicing are often the cause of human diseases.^28^, ^29^, ^30^ Although the majority of pathogenic variants can be detected with sequencing of the coding region, intronic variants may be missed because they are not captured in next‐generation sequencing studies, or because they are overlooked or filtered out during the sequence analysis. In this case, the variant was clearly present in exome sequencing data; however, a secondary analysis was required to identify the pathogenic variant. For the secondary analysis, we performed a detailed review of noncoding variants. In this case, the primary analysis was performed by a clinical sequencing laboratory and excluded consideration of deeper intronic variants. Further cDNA sequencing and/or WES may be required to identify unexpected deleterious variants that affect splicing.

Our study shows that sequence analysis strategies limited to analysis of coding region changes may miss pathogenic variants in known disease genes, especially if the variants are in noncoding regions. Although the first step in the analysis of osteogenesis imperfecta should include consideration of previously described mutations and detected coding variants in known disease genes, if the cause remains uncertain, we recommend reanalysis of sequencing data for rare variants that are present in known disease genes and functional characterization of those variants. This study shows that an integrated approach utilizing deep sequencing, informatics predictions, and functional characterization of suspicious noncoding variants may be required for pathogenic variant identification, in cases without identified pathogenic protein‐coding variants.

## Disclosures

All authors state that they have no conflicts of interest.

## Supporting information

Supporting Data S1.

## References

[1] Sykes B , Ogilvie D , Wordsworth P , et al. Consistent linkage of dominantly inherited osteogenesis imperfecta to the type I collagen loci: COL1A1 and COL1A2. Am J Hum Genet. 1990;46(2):293–307. 1967900 PMC1684971

[2] Marlowe A , Pepin MG , Byers PH. Testing for osteogenesis imperfecta in cases of suspected non‐accidental injury. J Med Genet. 2002;39(6):382–6. 12070242 10.1136/jmg.39.6.382 PMC1735162

[3] Forlino A , Cabral WA , Barnes AM , Marini JC. New perspectives on osteogenesis imperfecta. Nat Rev Endocrinol. 2011;7(9):540–57. 21670757 10.1038/nrendo.2011.81 PMC3443407

[4] Bardai G , Moffatt P , Glorieux FH , Rauch F. DNA sequence analysis in 598 individuals with a clinical diagnosis of osteogenesis imperfecta: diagnostic yield and mutation spectrum. Osteoporos Int. 2016;27(12):3607–13. 27509835 10.1007/s00198-016-3709-1

[5] Glorieux FH , Ward LM , Rauch F , Lalic L , Roughley PJ , Travers R. Osteogenesis imperfecta type VI: a form of brittle bone disease with a mineralization defect. J Bone Miner Res. 2002;17(1):30–8. 11771667 10.1359/jbmr.2002.17.1.30

[6] Homan EP , Rauch F , Grafe I , et al. Mutations in SERPINF1 cause osteogenesis imperfecta type VI. J Bone Miner Res. 2011;26(12):2798–803. 21826736 10.1002/jbmr.487 PMC3214246

[7] Becker J , Semler O , Gilissen C , et al. Exome sequencing identifies truncating mutations in human SERPINF1 in autosomal‐recessive osteogenesis imperfecta. Am J Hum Genet. 2011;88(3):362–71. 21353196 10.1016/j.ajhg.2011.01.015 PMC3059418

[8] Venturi G , Gandini A , Monti E , et al. Lack of expression of SERPINF1, the gene coding for pigment epithelium‐derived factor, causes progressively deforming osteogenesis imperfecta with normal type I collagen. J Bone Miner Res. 2012;27(3):723–8. 22113968 10.1002/jbmr.1480

[9] Filleur S , Nelius T , de Riese W , Kennedy RC. Characterization of PEDF: a multi‐functional serpin family protein. J Cell Biochem. 2009;106(5):769–75. 19180572 10.1002/jcb.22072

[10] Farber CR , Reich A , Barnes AM , et al. A novel IFITM5 mutation in severe atypical osteogenesis imperfecta type VI impairs osteoblast production of pigment epithelium‐derived factor. J Bone Miner Res. 2014;29(6):1402–11. 24519609 10.1002/jbmr.2173 PMC4352343

[11] Tucker T , Nelson T , Sirrs S , et al. A co‐occurrence of osteogenesis imperfecta type VI and cystinosis. Am J Med Genet A. 2012;158A(6):1422–6. 22528245 10.1002/ajmg.a.35319

[12] Rauch F , Husseini A , Roughley P , Glorieux FH , Moffatt P. Lack of circulating pigment epithelium‐derived factor is a marker of osteogenesis imperfecta type VI. J Clin Endocrinol Metab. 2012;97(8):E1550–6. 22669302 10.1210/jc.2012-1827

[13] Stephen J , Girisha KM , Dalal A , et al. Mutations in patients with osteogenesis imperfecta from consanguineous Indian families. Eur J Med Genet. 2015;58(1):21–7. 25450603 10.1016/j.ejmg.2014.10.001

[14] Minillo RM , Sobreira N , de Faria Soares Mde F, et al. Novel deletion of SERPINF1 causes autosomal recessive osteogenesis imperfecta type VI in two Brazilian families. Mol Syndromol. 2014;5(6):268–75. 25565926 10.1159/000369108 PMC4281578

[15] Caparros‐Martin JA , Valencia M , Pulido V , et al. Clinical and molecular analysis in families with autosomal recessive osteogenesis imperfecta identifies mutations in five genes and suggests genotype‐phenotype correlations. Am J Med Genet A. 2013;161A(6):1354–69. 23613367 10.1002/ajmg.a.35938

[16] Al‐Jallad H , Palomo T , Roughley P , et al. The effect of SERPINF1 in‐frame mutations in osteogenesis imperfecta type VI. Bone. 2015;76:115–20. 25868797 10.1016/j.bone.2015.04.008

[17] Ward L , Bardai G , Moffatt P , et al. Osteogenesis imperfecta type VI in individuals from Northern Canada. Calcif Tissue Int. 2016;98(6):566–72. 26815784 10.1007/s00223-016-0110-1

[18] Yang Y , Muzny DM , Reid JG , et al. Clinical whole‐exome sequencing for the diagnosis of Mendelian disorders. N Engl J Med. 2013;369(16):1502–11. 24088041 10.1056/NEJMoa1306555 PMC4211433

[19] Campeau PM , Lu JT , Sule G , et al. Whole‐exome sequencing identifies mutations in the nucleoside transporter gene SLC29A3 in dysosteosclerosis, a form of osteopetrosis. Hum Mol Genet. 2012;21(22):4904–9. 22875837 10.1093/hmg/dds326 PMC3607481

[20] Reid JG , Carroll A , Veeraraghavan N , et al. Launching genomics into the cloud: deployment of Mercury, a next generation sequence analysis pipeline. BMC Bioinformatics. 2014;15:30. 24475911 10.1186/1471-2105-15-30 PMC3922167

[21] Rimmer A , Phan H , Mathieson I , et al. Integrating mapping‐, assembly‐ and haplotype‐based approaches for calling variants in clinical sequencing applications. Nat Genet. 2014;46(8):912–8. 25017105 10.1038/ng.3036 PMC4753679

[22] 1000 Genomes Project Consortium; Auton A , Brooks LD , Durbin RM, et al. A global reference for human genetic variation. Nature. 2015;526(7571):68 –74. 26432245 10.1038/nature15393 PMC4750478

[23] Lek M , Karczewski KJ , Minikel EV , et al. Analysis of protein‐coding genetic variation in 60,706 humans. Nature. 2016;536(7616):285–91. 27535533 10.1038/nature19057 PMC5018207

[24] Wang K , Li M , Hakonarson H . ANNOVAR: functional annotation of genetic variants from high‐throughput sequencing data. Nucleic Acids Res. 2010;38(16):e164. 20601685 10.1093/nar/gkq603 PMC2938201

[25] Glorieux FH , Travers R , Taylor A , et al. Normative data for iliac bone histomorphometry in growing children. Bone. 2000;26(2):103–9. 10678403 10.1016/s8756-3282(99)00257-4

[26] Sule G , Campeau PM , Zhang VW , et al. Next‐generation sequencing for disorders of low and high bone mineral density. Osteoporos Int. 2013;24(8):2253–9. 23443412 10.1007/s00198-013-2290-0 PMC3709009

[27] Ogata N , Matsuoka M , Matsuyama K , et al. Plasma concentration of pigment epithelium‐derived factor in patients with diabetic retinopathy. J Clin Endocrinol Metab. 2007;92(3):1176–9. 17213275 10.1210/jc.2006-2249

[28] Teraoka SN , Telatar M , Becker‐Catania S , et al. Splicing defects in the ataxia‐telangiectasia gene, ATM: underlying mutations and consequences. Am J Hum Genet. 1999;64(6):1617–31. 10330348 10.1086/302418 PMC1377904

[29] Ars E , Serra E , Garcia J , et al. Mutations affecting mRNA splicing are the most common molecular defects in patients with neurofibromatosis type 1. Hum Mol Genet. 2000;9(2):237–47. 10607834 10.1093/hmg/9.2.237

[30] Faustino NA , Cooper TA. Pre‐mRNA splicing and human disease. Genes Dev. 2003;17(4):419–37. 12600935 10.1101/gad.1048803

