# Supplementary material for: Whole‐Exome Sequencing Identifies an Intronic Cryptic Splice Site in SERPINF1 Causing Osteogenesis Imperfecta Type VI
Source: JBMR Plus. 2018 Apr 16;2(4):235–9. doi: 10.1002/jbm4.10044 (PMC6124173; doi:10.1002/jbm4.10044)
Supplement: Supplementary file 1 — Supporting Data S1. [file JBM4-2-235-s001.docx]

Supplemental data

Supplementary Table 1. Primers list.

| **Name** | **Experiment** | **Primer sequence (5’-3’)** | **Product size(bp)** |
| --- | --- | --- | --- |
| P1-F | cDNA analysis | GGTCGCTTTAAGAAAGGAGTAGCTGT | 413 |
| P1-R |  | CCACACTGAGAGGAGACAGGAGCACGTT |  |
| P2-F | cDNA analysis | AGGCCCTGGTGCTACTCCTCTGCATT | 593 (control splicing);  625 (proband alternative splicing) |
| P2-R |  | AATTTCCTTTGTGGACCTGGCGAGCTT |  |
| P3-F | cDNA analysis | TGAAAGGGAAGCTCGCCAGGTCCACA | 350 |
| P3-R |  | TTCTCGGTCTATGTCATGAATGAACTCGG |  |
| P4-F | cDNA analysis | ATGTCGGACCCTAAGGCTGTTTTACGC | 576 |
| P4-R |  | CTGTCCCTCGGGTTTTCTTCTAGGGTA |  |
| Seq-F | Genome analysis | ATTCACCGGGCTCTCTACTA | 418 |
| Seq-R |  | AGGTCAGGAGATCGAGACTATC |  |
